# Gastrointestinal stromal tumors in Japanese patients with neurofibromatosis type I

**DOI:** 10.1007/s00535-015-1132-6

**Published:** 2015-10-29

**Authors:** Toshirou Nishida, Masahiko Tsujimoto, Tsuyoshi Takahashi, Seiichi Hirota, Jean-Yves Blay, Mari Wataya-Kaneda

**Affiliations:** Department of Surgery, Osaka Police Hospital, Osaka, Japan; Department of Pathology, Osaka Police Hospital, Osaka, Japan; Department of Surgery, National Cancer Center Hospital East, 6-5-1 Kashiwanoha, Kashiwa, Chiba 277-8577 Japan; Department of Surgery, Osaka University Graduate School of Medicine, Osaka, Japan; Department of Surgical Pathology, Hyogo College of Medicine, Nishinomiya, Hyogo Japan; Centre Leon Bernard of the Université Claude Bernard, Lyon, France; Department of Dermatology, Osaka University Graduate School of Medicine, Osaka, Japan

**Keywords:** Neurofibromatosis type I, Gastrointestinal stromal tumor, Prognosis, Clinicopathologic features

## Abstract

**Background:**

Neurofibromatosis type I (NF1) predisposes patients to various neoplasias, including gastrointestinal stromal tumors (GISTs). Little is known about the risk of developing GISTs for NF1 patients or the clinicopathologic features and prognosis of NF1-GIST.

**Methods:**

We conducted a multi-detector computed tomography screen for adult NF1 patients between 2003 and 2012. Clinicopathologic data of sporadic GISTs from patients who underwent surgery between 2001 and 2010 were retrospectively collected from 32 hospitals in Japan.

**Results:**

CT screening identified 6 GIST patients from the 95 NF1 patients screened, suggesting that the prevalence rate of GISTs was approximately 6.3/100 in NF1 patients. All 6 NF1 patients exhibited hyperplasia of the interstitial cells of Cajal in the adjoining small intestine. NF1-GISTs may account for 1.1–1.3 % of primary sporadic GISTs and present as multiple tumors in the small intestine, with low mitotic activity and no *KIT* or *PDGFRA* mutations. The risk of recurrence and mortality is very similar between NF1 and non-NF1 patients after surgical resection of GISTs.

**Conclusions:**

NF1 patients may be predisposed to developing small intestinal GISTs, which may appear as multiple GISTs without *KIT* and *PDGFRA* mutations. The prognosis of patients with NF1-GISTs is similar to patients with conventional GISTs.

**Electronic supplementary material:**

The online version of this article (doi:10.1007/s00535-015-1132-6) contains supplementary material, which is available to authorized users.

## Introduction

Gastrointestinal stromal tumors (GISTs) predominantly occur in the stomach (60–70 %) and small intestine (20–30 %), and multiple tumors are rarely observed [1, 2]. GIST proliferation may be caused by gain-of-function mutations in either the *KIT* (80–85 %) or *PDGFRA* (10 %) genes [2]. Five to ten percent of GISTs lack mutations in these genes (wild-type GISTs). Such wild-type GISTs are now known to be heterogeneous and may actually possess mutations in the *SDH* complex genes, *NF1*, *BRAF*, *NRAS*, or *HRAS*. Imatinib mesylate, a selective tyrosine kinase inhibitor of KIT, PDGFR-α, and Abl, exhibits exceptional activity in advanced GIST patients. The benefits of imatinib have largely been observed in *KIT*- and *PDGFRA*-mutant GISTs; however, its activities are less well documented in wild-type GISTs [2].

Neurofibromatosis type I (NF1), also known as von Recklinghausen disease, is an autosomal dominant inherited syndrome affecting 1/3000–4000 individuals [3]. NF1 is distinguished by a variety of characteristic features and is clinically diagnosed using the National Institutes of Health diagnostic criteria [4]. NF1 is believed to be caused by functionally biallelic losses of the tumor suppressor gene *NF1*, resulting in the functional loss of neurofibromin. NF1 patients are predisposed to both benign and malignant tumors, including neurofibroma, optic-pathway glioma, malignant peripheral nerve-sheath tumors (MPNSTs), neuroblastoma, GIST, pheochromocytoma, and breast cancer [3].

A number of case reports and small case series have indicated a strong association between NF1 and GIST [3, 5].The clinicopathologic features of GISTs associated with NF1 are different from those of sporadic GISTs. Most NF1 patients, for instance, exhibit multiple GISTs in the small intestine, and NF1-GISTs rarely exhibit mutations in the *KIT* and *PDGFRA* genes [3, 7]. However, the existing studies of NF1-GISTs have been primarily conducted in Caucasian populations and are mostly case series with small numbers of patients or case reports. Some conflicting results were observed from these reports, [9, 10] and the incidence of GISTs in Japanese NF1 patients and the clinicopathologic features of NF1-GISTs in a Japanese population have not yet been established. Furthermore, to date, no report has demonstrated the outcomes of surgery and prognosis for NF1-GIST patients.

Although NF1 may be associated with an increased risk for GIST, little is known about the lifetime risk of developing a GIST in NF1 patients. Moreover, the frequency of NF1-GIST development among sporadic GISTs and the clinicopathologic characteristics and prognosis of NF1-GIST remain unknown. In this study, we, therefore, evaluated NF1 patients by CT screening, and retrospectively analyzed the clinical and pathological characteristics of any identified NF1-GISTs. We also assessed NF1-GIST patient prognosis.

## Patients and methods

### NF1 patient screening with multi-detector CT (MDCT)

Because of the suspected high incidence of intestinal GISTs in adult NF1 patients, we planned and conducted multi-detector computed tomography (MDCT) screening. We recommended MDCT to adult NF1 patients over 30 years old who visited the Department of Dermatology at the Osaka University Hospital as well as to NF1 patients with signs and symptoms of the disease. The MDCT screening was offered to 158 NF1 patients between April 2003 and September 2012. Of these 158 NF1 patients, informed consent was obtained from 95, and they received MDCT with or without contrast media. We recommended the use of contrast media; however, when patients had a suspected allergy to the media or refused its use, a plain CT was performed. The details of the scanning protocol were similar to those described elsewhere [16]. The diagnosis of NF1 was confirmed according to the NIH diagnostic criteria [4]. Data were prospectively collected and retrospectively analyzed. This study was approved by the institutional review board of Osaka University Hospital and was conducted according to institutional ethics guidelines.

### Retrospective cohort study of sporadic GISTs

We retrospectively collected NF1-GISTs and sporadic GISTs from 29 community hospitals and 3 institutions in Japan between 2001 and 2010. We sent survey forms to 32 hospitals and institutes, which identified 23 primary NF1-GIST patients among 1314 patients with a primary GIST who underwent surgery during this period.

Clinical and pathological data for these NF1-GISTs were collected with tissue samples for pathological examination when available. Clinicopathologic data for sporadic GISTs other than those in NF1-GIST patients were obtained from 19 hospitals. Detailed clinicopathologic data were obtained from 927 of the 1314 primary sporadic GIST patients. This study was approved by the institutional review board of Osaka University Hospital and Osaka Police Hospital and was conducted according to institutional guidelines. NF1-GISTs were also identified from a database of reference centers for sarcoma in France (NetSARC, netsarc.org), which has now gathered over 21,000 sarcoma tumors since 2010. This database, updated in real-time by the 26 reference centers in France, collects data on an estimated 85 % of the sarcoma cases in this country.

### Pathological diagnosis and mutational analysis

Tissue samples fixed with 10 % buffered formalin and embedded in paraffin were sectioned (3 µm thick) and used for hematoxylin and eosin staining (H&E) and immunohistochemistry (IHC). IHC was performed using the ENVISION + KIT HRP (DAB) system (DAKO, Glostrup, Denmark) as previously described [17]. Histopathologic features, including cell shape, mean mitotic number per 50 HPF, and immunophenotype, were analyzed by H&E and IHC.

When freshly frozen samples were available, total RNA was extracted using the RNeasy Mini Kit (QIAGEN, Valencia, CA, USA). All coding regions of the *KIT* and/or *PDGFRA* genes were amplified by polymerase chain reaction (PCR) after reverse transcription of the extracted RNA as previously described [17, 18]. When only paraffin blocks were available, genomic DNA was extracted from the paraffin sections using the QIAamp DNA Mini Kit (QIAGEN). Exons 9, 11, 13, and 17 of the *KIT* gene and exons 12, 14, and 18 of the *PDGFRA* gene were amplified by PCR using previously described primers [17, 19].

### Statistical analysis

Statistical analyses were performed using the Chi squared test, Fisher’s exact test, Student’s *t* test, and the Mann–Whitney *U* test. The recurrence-free survival (RFS) was calculated from the date of surgery to the date of first tumor recurrence or to the date of death, censoring living patients without recurrence at the time of data collection. The overall survival (OS) was calculated from the date of surgery to the date of death, censoring living patients. RFS and OS between the groups were compared using the Kaplan–Meier life-table method with the log-rank test. The *p* values were two-sided, and *p* values <0.05 were considered significant. The data were analyzed using the Statistical Package for Social Sciences, version 17.0 (SPSS Inc., Chicago, IL, USA).

## Results

### MDCT screening of NF1 patients

Ninety-five NF1 patients, comprising 35 males and 60 females with a median age of 45 years (range 17–80), received MDCT with or without contrast media. One patient (a 66-year-old male) exhibited anemia in a pre-screening blood examination, but all other patients did not exhibit gastrointestinal symptoms or signs before screening. Radiographic findings of neoplastic lesions included 6 gastrointestinal tumors (6.3 %; CI: 2.35–13.2 %), which were later diagnosed as GISTs after pathological examination of surgical specimens, 6 myoma uteri, 3 adrenal tumors, 3 intrapelvic masses suspected to be neurofibromas, and 1 mediastinal tumor (Table 1).

**Table 1:** Abdominal lesions indicated by MDCT screening

Lesions	*N* (%)
GIST	6 (6.3 %; CI: 2.35–13.24)
Myoma uteri	6 (6.3 %, 10 % for female)
Adrenal tumor	3 (3.2 %)
Suspected pelvic neurofibroma	3 (3.2 %)
Mediastinal tumor	1 (1.0 %)
Gallstone	4 (4.2 %)
Renal artery aneurysm	1 (1.0 %)

**Fig. 1:**
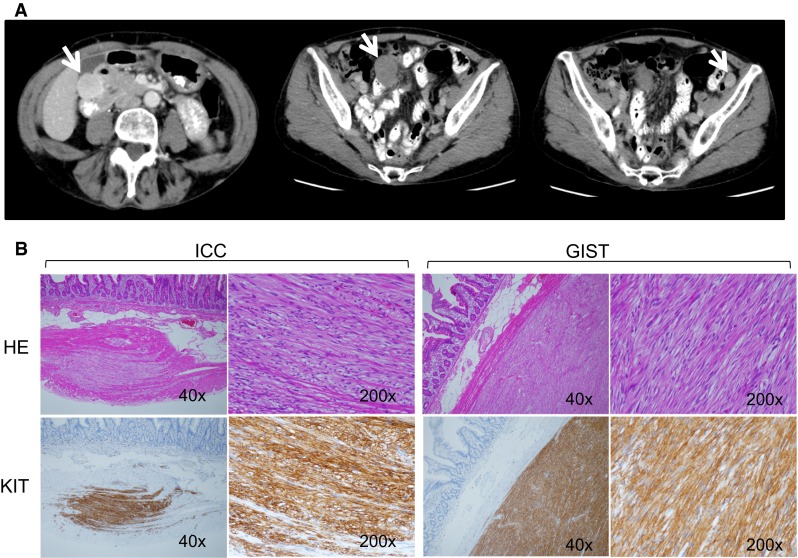
Screening NF-1 patients by MDCT. **a** Presents a representative case of an NF-1 patient whose multiple GISTs were detected by MDCT screening. The patient was a 64-year-old female without signs or symptoms. The *arrows* indicate multiple intestinal GISTs. **b** Demonstrates ICC hyperplasia (*left*) in the normal jejunum and a GIST (*right*) observed in an NF1 patient with GISTs. The upper figures show H&E staining, and the lower figures show KIT immunostaining. “×40” and “×200” indicate the magnification of the objective lens

All 6 NF1 patients with gastrointestinal tumors underwent surgery. At the time of surgery, 5 of the 6 NF1 patients had multiple GISTs in the duodenum, jejunum, and/or ileum, and 1 patient had a single tumor in the jejunum (Fig. 1a; Table 2). Pathologically, all resected tumors were KIT-positive, DOG1-positive spindle cell tumors with few mitotic figures. Furthermore, pathological examination revealed that all 6 NF1 patients exhibited hyperplasia of the interstitial cells of Cajal (ICC) in the normal-appearing adjoining small intestine (Fig. 1b). *KIT* and *PDGFRA* genetic analysis revealed that no resected tumors exhibited mutations in either gene (Table 2).

**Table 2:** Characteristic features of NF1-GISTs found by the MDCT screening

Age	Sex	Organs	Multiplicity	Max size (cm)	ICC hyperplasia	Cell type	Mitosis (/50HPF)	Mutations	
								*KIT*	*PDGFRA*
66	M	D, J	Yes	4	Yes	Spindle	0	None	None
60	M	J	No	6	Yes	Spindle	1	None	None
38	F	J	Yes	3	Yes	Spindle	0	None	None
51	M	D, J	Yes	1.8	Yes	Spindle	1	None	None
31	M	J, I	Yes	3	Yes	Spindle	0	None	None
64	F	D, J	Yes	4.2	Yes	Spindle	0	None	None

*D* duodenum, *J* jejunum, *I* ileum

### NF1-GISTs represent a rare sub-fraction of sporadic GISTs

Next, we collected histologically confirmed GISTs, including NF1-GISTs, from patients who underwent surgery between 2001 and 2010 at 32 hospitals in Japan. In total, 1314 primary GISTs were collected, including 23 primary NF1-GISTs (1.75 %; CI: 1.17–2.61 %). Six of the 23 NF1-GISTs were detected by CT screening, and when these 6 NF1-patients were excluded from the analysis, NF1-GISTs accounted for 1.30 % (CI: 0.81–2.07 %) of the sporadic GISTs. A similar investigation was performed using data obtained from the French Netsarc/Rreps networks study. In this registry, 18 NF1-GIST patients (1.1 %; CI: 0.75–1.85 %) were identified among 1528 GIST cases. Thus, NF1-GISTs are estimated at 1.1–1.3 % of primary sporadic GISTs.

Clinicopathologic features were compared between the non-NF1-GISTs and NF1-GISTs (Table 3). The NF1-GISTs more frequently presented as multiple lesions preferentially located in the small intestine compared with those of the non-NF1-GISTs. R0 or R1 surgery was less frequently performed for NF1-GIST patients than for non-NF1-GIST patients. The mitotic activity of the NF1-GISTs was significantly lower for mitotic counts (/50 HPF) and Ki67-positive tumor cells than that of the non-NF1-GISTs. *KIT* or *PDGFRA* mutations were not observed in the studied NF1-GISTs while 294 of the 303 non-NF1-GISTs (97.0 %) exhibited *KIT* or *PDGFRA* mutations. In pathological examinations, NF1-GIST cells appeared more spindle-shaped, but this finding was not statistically significant. Thus, no significant differences were observed between the 2 populations with respect to their age, gender, tumor size, recurrence, cell type, or KIT-immunoreactivity.

**Table 3:** Clinicopathologic features of NF-1 GISTs and sporadic GISTs

	NF-1 GIST		Sporadic GIST	*p* values^a^
	Japanese set	French set	Japanese set	
No. of NF1-GISTs	22	18	904	
Median follow-up (yrs)	3.3		3.6	
Median age at d*x*	61 (26–77)	51 (30–67)	64 (10–93)	0.4302
Gender				
Male	9	6	481	0.3543
Female	13	12	423	
Median size (cm)	4.1 (1.8–21)	6.6 (1.7–31)	5.0 (0.7–35)	0.5519
Multiplicity				
No	6	0	867	<0.0001
Yes	16	0	17	
Not available	0	18	20	
Location				
Esophagus	0	0	9	<0.0001
Stomach	0	1	623	
Intestine	22	15	213	
Colon	0	0	52	
Others	0	2	7	
Curability^b^				
R0, R1	15	7	784	0.0119^d^
R2	7	1	104	
Not available	0	10	16	
Mutations^c^				
Yes	0	0	294	<0.0001
No	8	6	9	
Not available	14	12	601	
Recurrence				
No	18	8	679	0.6382^d^
Yes	4	1	225	
Not available	0	9	0	
Prognosis (OS)				
Alive	20	11	734	0.3790^d^
Dead	2	0	170	
Not available	0	7	0	
Mitosis (/50HPF)	0.0 (0–8)	1 (0–3)	3.0 (0–300)	0.0093
	(*n* = 22)	(*n* = 3)	(*n* = 902)	
Ki67 (%)	0.5 (0.5–15)	NA	2.5 (0–50)	0.0022
	(*n* = 21)		(*n* = 248)	
Cell type				
Spindle	21	4	671	0.0636
Epithelioid or mixed	1	0	75	
Not available	0	14	158	

*NA* not available

^a^Comparison between Japanese NF-1 GISTs and Japanese sporadic GISTs

^b^Curability of surgery

^c^Mutations in the *KIT* and *PDGFRA* genes

^d^ By the Fisher’s exact test

**Fig. 2:**
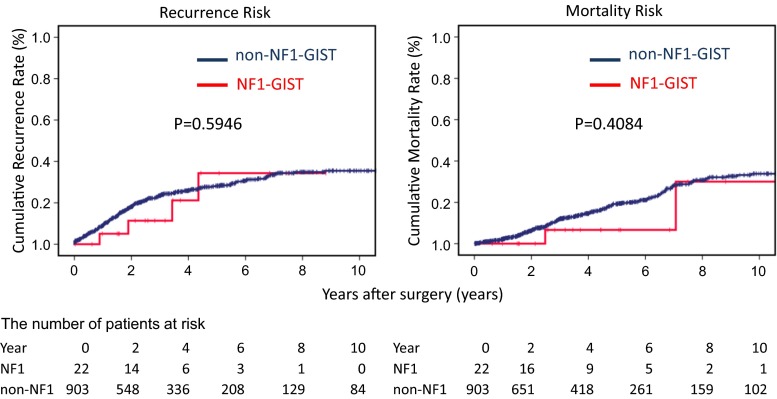
NF1-GIST patient prognosis. The cumulative recurrence rate (*right panel*) and cumulative mortality rate (*left panel*) are shown. The median RFS was not reached in the NF1-GIST group, but the median RFS in the non-NF1-GIST group was estimated at 19.0 years. Median OS was not reached in both groups

With a median follow-up of 3.6 years, 4 NF1-GIST patients (18.2 %) had relapses in their GISTs, and 225 non-NF1 (24.9 %) patients had recurrences (Table 3). Peritoneal recurrence was the primary relapse site for all 4 NF1-GIST patients. Among the 7 NF1-GIST patients who underwent R2 surgery, only 2 patients had recurrences during a median follow-up of 4.3 years. No significant difference was observed in recurrence risk between the two groups (*p* = 0.5946). Two of the 4 recurrent NF1-GIST patients received imatinib (400 mg/day), and both showed disease progression after 3 months of therapy. Thereafter, both patients received sunitinib (50 mg/day). One patient showed progressive disease (PD) after 4.5 months, and the other was diagnosed with PD after 3 months. The mortality risk was not different between the NF1-GIST patients and the non-NF1-GIST patients (*p* = 0.4084): 2 patients (9.1 %) of the 22 NF1-GIST patients died during follow-up, while 170 of the non-NF1 patients with sporadic GISTs (18.8 %) died during follow-up (Table 3; Fig. 2). One of the NF1-GIST patients died from disease progression, and the other died following lung cancer. Because the risk of recurrence is higher in small intestinal GISTs compared with gastric GISTs [1, 2] and because all NF1-GISTs in this study were located in the small intestine, we further examined RFS and OS in NF1-GIST and non-NF1 patients with small intestinal GISTs (Supplemental Table 1). The prognosis of the NF1-GIST patients was significantly better for RFS (*p* = 0.0383) and marginally better for OS (*p* = 0.0776) than for the non-NF1 patients with small intestinal GISTs (Supplemental Fig. 1). However, the number of events was limited.

## Discussion

Although several retrospective studies have reported an increased risk for GISTs in NF1 patients, most reports have suffered from small sample sizes. Furthermore, the frequency of GISTs in NF-1 patients varies greatly in the literature, ranging from 5 to 29 % [3, 5–8, 10, 11, 14, 20]. One epidemiologic study using the Swedish Cancer Registry estimated that the lifetime risk of an NF1 patient developing GISTs could be as high as 7 % [3, 11]. The clinical incidence of GISTs, and particularly intestinal GISTs, may vary, as suggested by the number of asymptomatic GISTs identified and studied here. Indeed, NF1-GIST incidence may have been estimated to be higher in a report where half of the NF1-GISTs were incidentally discovered [14], whereas another report estimating a lower incidence involved predominantly NF1-GIST patients with gastrointestinal symptoms [8]. Furthermore, all these reports retrospectively collected NF1-GISTs. In the present study, we prospectively examined the intra-abdominal tumors of NF-1 patients by MDCT and observed that 6 of 95 NF1 patients exhibited small-intestinal GISTs. Furthermore, 5 of 6 patients were asymptomatic, suggesting that a significant number of NF1 patients in middle age could potentially have subclinical intestinal GISTs. The prevalence rate is 6.3/100 NF1 patients and is similar to that obtained by the Swedish Cancer Registry [11]. In our study, the incidence rate was estimated at 6.3/1000/NF1 patients/year, and the target NF1 patients were typically between 30 and 60 years old. Thus, GIST incidence in the NF1 patients was estimated at 200-fold higher than that for sporadic GISTs, which was postulated at 15 cases per year per million individuals. Although the usefulness of MDCT screening for intestinal tumors has not been established, MDCT can detect intestinal masses larger than 2 cm [16, 21].

From our study, NF1-GISTs accounted for 1–2 % of all GISTs, which is consistent with previous reports [8, 14]. Thus, the incidence rate and the proportion of NF1-GISTs in sporadic GISTs appear very similar in Japanese and Caucasian populations. Most NF1-GISTs are located in the small intestine, including the duodenum and proximal jejunum, and frequently present as multiple tumors with an indolent nature. Contrary to our observations, some studies indicated that the age of GIST diagnosis tends to be younger for NF1 patients than for patients with sporadic GISTs [8, 14]. The mitotic counts for NF1-GISTs were lower than those for sporadic GISTs in this study; however, the observed tumor size was similar, contradicting the results of a previous study without a control arm [8]. Many reports, including this study, have observed the majority of NF1-GISTs lack *KIT* and *PDGFRA* mutations, [3, 6–10, 12–15, 22] and accordingly, NF1-GISTs might not respond to imatinib [10].

Neurofibromin is a negative regulator of Ras kinases, and its loss of function may activate Ras and downstream kinases, including those in the MEK-MAPK pathway [3]. The MEK-MAPK pathway and the subsequent expression of ETV1, a master regulator of an ICC-GIST-specific transcription network and KIT expression [23], is predominantly activated in NF1-GISTs [15], which may result in strong KIT-positivity in immunohistochemistry. In this study, NF1 patients with GISTs exhibited ICC hyperplasia in the myenteric plexus of the small intestine, as observed in familial GIST patients with germline *KIT* mutations [24]. More than half of NF1-GIST patients exhibited multiple GISTs in the small intestine [8, 10]. These observations may be related to the high incidence of GISTs in NF1 patients. A previous report investigating clonality in familial GIST patients with germline *KIT* mutations indicated that cells in the ICC hyperplasia have polyclonal proliferation, whereas GIST tumor cells show a monoclonal development [25].

This study indicated that NF1-GIST patients underwent more frequent R2 surgery than non-NF1-GIST patients and that NF1-GIST relapses were mainly peritoneal disease (Table 3). These results suggest that multiple occurrences of intestinal GISTs may be responsible for incomplete surgery and peritoneal recurrence in NF1 patients. However, the recurrence rate in NF1 patients was similar to that of non-NF1-GIST patients (Fig. 2). The OS of NF1-GIST patients was not inferior to that of sporadic GIST patients, although imatinib failed to show any activity on recurrent NF1-GISTs [10]. Furthermore, when the prognosis was compared with the condition of small-intestinal GISTs, the RFS and OS of the NF1-GIST patients appeared superior to those of the non-NF1-GIST patients (Supplemental Fig. 1). These results could be from the indolent nature and low proliferation activity of the NF1-GISTs [7, 10]. In fact, the mitotic count and Ki67 staining of the NF1-GISTs were significantly lower than those of the other sporadic GISTs, and 5 NF1-GIST patients with R2 surgery had no evidence of recurrence at the time of analysis, with a median follow-up of 2.5 years.

In summary, we have prospectively evaluated the incidence of GISTs in NF-1 patients. The prevalence rate of GISTs was estimated at nearly 6 % in adult NF1 patients, and NF1-GISTs may account for 1–2 % of total sporadic GISTs. The risk of developing GISTs in NF1 patients is estimated at nearly 200-fold higher than that in normal populations. The clinical, pathologic and genetic features of NF1-GISTs differ from those of sporadic GISTs, including the development of multiple small intestinal tumors, an absence of *KIT* and *PDGFRA* mutations, and an indolent nature. Although the NF1-GISTs are frequently accompanied with multiple tumors and R2 surgery, the RFS and OS of NF1-GIST patients are similar to those of the normal population with sporadic GISTs.

## Electronic supplementary material

Below is the link to the electronic supplementary material.

Supplementary material 1 (DOCX 19 kb)

Supplementary material 2 (PPTX 129 kb)

## Abbreviations

GIST: Gastrointestinal stromal tumor
IHC: Immunohistochemistry
ICC: Interstitial cells of Cajal
MDCT: Multi-detector computed tomography
MPNST: Malignant peripheral nerve-sheath tumors
NF1: Neurofibromatosis type I
NF1-GIST: GIST associated with NF1
OS: Overall survival
PD: Progressive disease
RFS: Recurrence-free survival
SDH: Succinate dehydrogenase
