# Supplementary material for: Gastrointestinal stromal tumors in Japanese patients with neurofibromatosis type I
Source: J Gastroenterol. 2015 Oct 29;51:571–8. doi: 10.1007/s00535-015-1132-6 (PMC4880630; doi:10.1007/s00535-015-1132-6)
Supplement: Supplementary file 1 — Supplementary material 1 (DOCX 19 kb) [file 535_2015_1132_MOESM1_ESM.docx]

Supplemental Table 1. Comparison of clinicopathologic features of NF-1 GISTs and sporadic small intestinal GISTs

NF-1 GIST Sporadic small intestinal GIST p values^#^

No. of NF1-GISTs 22 213

Median follow-up (yrs) 3.3 3.5

Median age at dx 61 (26-77) 60 (16-91) 0.6061

Gender: Male 9 122 0.2137

Female 13 91

Median size (cm) 4.1 (1.8-21) 7.0 (0.7-30) 0.0503

Multiplicity No 6 204 <0.0001

Yes 16 6

Not available 0 20

Curability* R0, R1 15 148 0.8847

R2 7 63

Not available 0 2

Mutations^&^ Yes 0 80 <0.0001

No 8 0

Not available 14 130

Recurrence No 18 115 0.0128^$^

Yes 4 98

Prognosis (OS) Alive 20 137 0.0153^$^

Dead 2 76

Mitosis (/50HPF) 0.0 (0-8) 5.0 (0-187) <0.0001

(n=22) (n=212)

Ki67 (%) 0.5 (0.5-15) 3.0 (0-50) <0.0001

(n=21) (n=69)

Cell type

Spindle 21 163 0.1021

Epithelioid or mixed 1 21

Not available 0 158

*: curability of surgery

&: mutations in the *KIT* and *PDGFRA* genes

$: by the Fisher’s exact test
