# Supplementary material for: Gastrointestinal stromal tumors in Japanese patients with neurofibromatosis type I
Source: J Gastroenterol. 2015 Oct 29;51:571–8. doi: 10.1007/s00535-015-1132-6 (PMC4880630; doi:10.1007/s00535-015-1132-6)
Supplement: Supplementary file 2 — Supplementary material 2 (PPTX 129 kb) [file 535_2015_1132_MOESM2_ESM.pptx]

## Slide 1
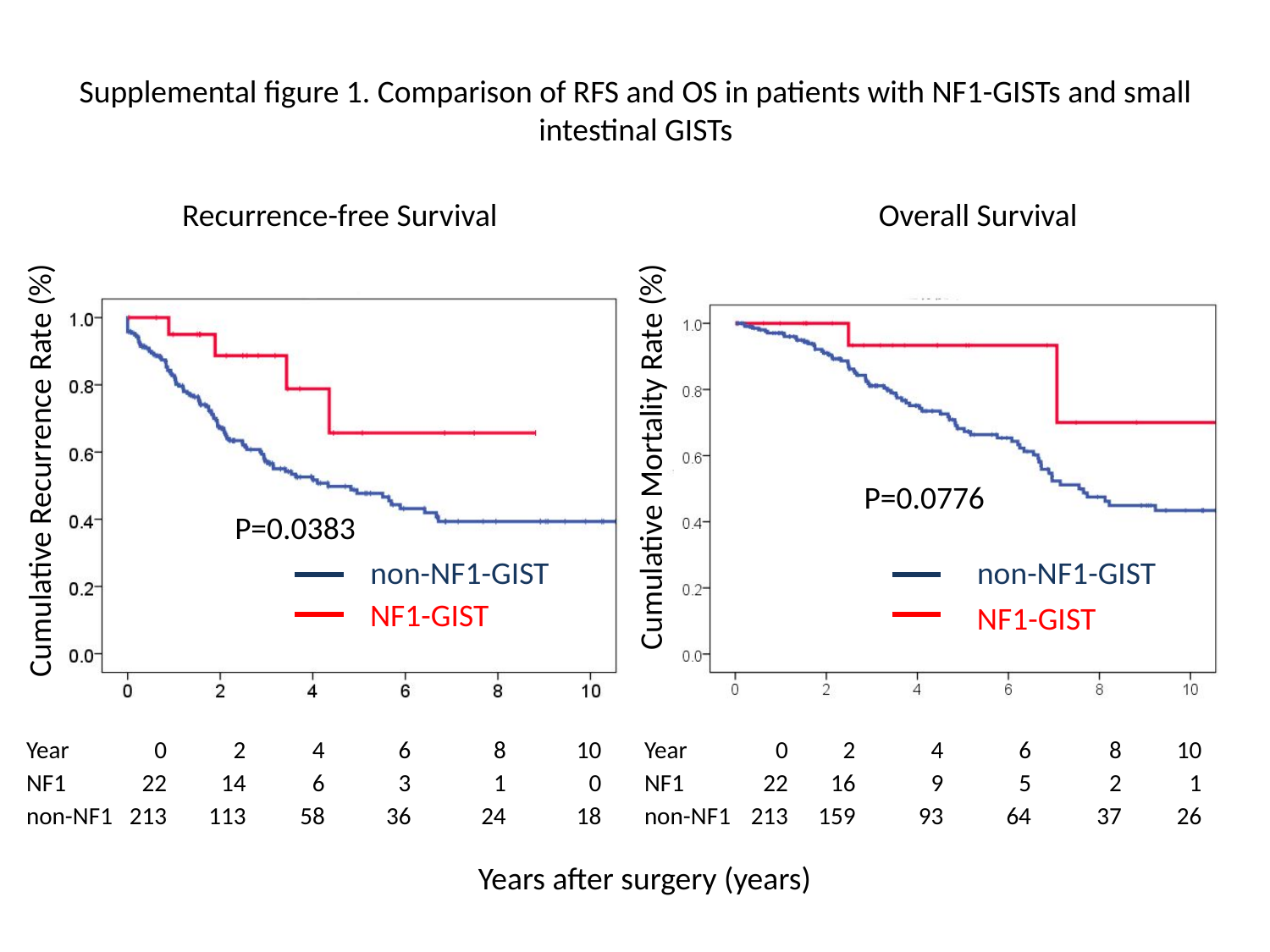

# Supplemental figure 1. Comparison of RFS and OS in patients with NF1-GISTs and small intestinal GISTs
Recurrence-free Survival
Overall Survival
Cumulative Mortality Rate (%)
Cumulative Recurrence Rate (%)
P=0.0776
P=0.0383
non-NF1-GIST
non-NF1-GIST
NF1-GIST
NF1-GIST
| Year | 0 | 2 | 4 | 6 | 8 | 10 |
| --- | --- | --- | --- | --- | --- | --- |
| NF1 | 22 | 14 | 6 | 3 | 1 | 0 |
| non-NF1 | 213 | 113 | 58 | 36 | 24 | 18 |
| Year | 0 | 2 | 4 | 6 | 8 | 10 |
| --- | --- | --- | --- | --- | --- | --- |
| NF1 | 22 | 16 | 9 | 5 | 2 | 1 |
| non-NF1 | 213 | 159 | 93 | 64 | 37 | 26 |
Years after surgery (years)
